# Reduced Renal α-Klotho Expression in CKD Patients and Its Effect on Renal Phosphate Handling and Vitamin D Metabolism

**DOI:** 10.1371/journal.pone.0086301

**Published:** 2014-01-23

**Authors:** Hirokazu Sakan, Kimihiko Nakatani, Osamu Asai, Akihiro Imura, Tomohiro Tanaka, Shuhei Yoshimoto, Noriyuki Iwamoto, Norio Kurumatani, Masayuki Iwano, Yo-ichi Nabeshima, Noboru Konishi, Yoshihiko Saito

**Affiliations:** 1 First Department of Internal Medicine, Nara Medical University, Kashihara, Nara, Japan; 2 Department of Pathology, Nara Medical University, Kashihara, Nara, Japan; 3 Department of Pathology and Tumor Biology, Kyoto University Graduate School of Medicine, Kyoto, Japan; 4 Department of Urology, Tojinkai Hospital, Kyoto, Japan; 5 Departments of Community Health and Epidemiology, Nara Medical University, Kashihara, Nara, Japan; 6 Division of Nephrology, Department of General Medicine, Faculty of Medical Sciences, University of Fukui, Yoshida-gun, Fukui, Japan; 7 Department of Regulatory Medicine for Blood Pressure, Nara Medical University, Kashihara, Nara, Japan; Institut National de la Santé et de la Recherche Médicale, France

## Abstract

Renal α-Klotho (α-KL) plays a fundamental role as a co-receptor for fibroblast growth factor 23 (FGF23), a phosphaturic hormone and regulator of 1,25(OH)_2_ vitamin D_3_ (1,25VitD_3_). Disruption of FGF23-α-KL signaling is thought to be an early hallmark of chronic kidney disease (CKD) involving reduced renal α-KL expression and a reciprocal rise in serum FGF23. It remains unclear, however, whether the rise in FGF23 is related to the loss of renal α-KL. We evaluated α-KL expression in renal biopsy samples and measured levels of several parameters of mineral metabolism, as well as soluble α-KL (sKL), in serum and urinary samples from CKD patients (n = 236). We found that although renal α-KL levels were significantly reduced and serum FGF23 levels were significantly elevated in early and intermediate CKD, serum phosphate levels remained within the normal range. Multiple regression analysis showed that the increases in FGF23 were significantly associated with reduced renal function and elevated serum phosphate, but were not associated with loss of renal α-KL. Moreover, despite falling renal α-KL levels, the increase in FGF23 enhanced urinary fractional excretion of phosphate and reduced serum 1,25VitD_3_ levels in early and intermediate CKD, though not in advanced CKD. Serum sKL levels also fell significantly over the course of CKD, and renal α-KL was a significant independent determinant of sKL. These results demonstrate that FGF23 levels rise to compensate for renal failure-related phosphate retention in early and intermediate CKD. This enables FGF23-α-KL signaling and a neutral phosphate balance to be maintained despite the reduction in α-KL. In advanced CKD, however, renal α-KL declines further. This disrupts FGF23 signaling, and serum phosphate levels significantly increase, stimulating greater FGF23 secretion. Our results also suggest the serum sKL concentration may be a useful marker of renal α-KL expression levels.

## Introduction

α-Klotho (α-KL) is a single-pass transmembrane protein [1], [2] expressed in multiple tissues, but present at particularly high levels in the kidney. It was originally described as a senescence-related protein because mice lacking functional α-KL protein develop a syndrome resembling human aging [1]. Recently, however, α-KL was shown to act as a co-receptor that forms a complex with fibroblast growth factor receptor 1 (FGFR1) to mediate signaling by the circulating hormone fibroblast growth factor 23 (FGF23), which is an important regulator of mineral metabolism [3], [4]. Within the kidney, FGF23 activity leads to phosphaturia and down-regulation of renal 1,25-dihydroxy vitamin D3 (1,25VitD_3_) production [5], [6]. The importance of α-KL for FGF23 signaling in the kidney is apparent in *Klotho*- and *Fg23*-null mice, which share nearly identical biochemical phenotypes that are consistent with the dismantling of FGF23 signaling, including hyperphosphatemia and elevated 1,25VitD_3_ [1], [7].

Notably, recent studies have shown that serum FGF23 levels gradually increase during the progression of chronic kidney disease (CKD), whereas renal α-KL expression declines [8], [9], [10]. CKD is the most common cause of chronically elevated FGF23 levels, which independently associate with CKD progression, the occurrence of cardiovascular events and mortality among CKD populations [11], [12], [13], [14]. The specific cause of the elevation in circulating FGF23 seen in CKD patients remains unclear, but one potential candidate is loss of renal α-KL expression, since the resultant α-KL deficiency could make the kidney resistant to the action of FGF23. To date, however, there have been no studies characterizing the relation between serum FGF23 levels and renal α-KL levels in CKD patients

In addition to the membrane-anchored form of α-KL, a soluble form produced through alternative splicing of the α-KL transcript [15], [16] or ectodomain shedding catalyzed by desintegrin and metalloproteinase [17], [18] was recently detected in cerebrospinal fluid, blood, and urine [19]. Soluble α-KL (sKL) is a pleiotropic protein functioning as an endocrine factor with multiple renal and extrarenal effects. Previous studies have suggested that circulating sKL may represent a useful biomarker for diagnosis of CKD [10]. However, the relation between circulating sKL and renal α-KL in CKD patients has not yet been characterized.

The purpose of the present study was to determine whether renal α-KL levels modulate serum levels of FGF23 and sKL, and to assess the potential role of renal α-KL in the mineral and bone disorders seen in CKD patients. To that end, we analyzed the association between renal α-KL expression and several parameters of mineral metabolism, including FGF23 and sKL levels in serum collected from CKD patients, who also provided renal biopsy samples. In addition, we examined the effects of α-KL deficiency on FGF23 signaling in cultured human embryonic kidney 293 (HEK293) cells.

## Materials and Methods

### Study population

This study evaluated 236 patients who had undergone renal biopsy for CKD. Measured in all patients were serum levels of total protein, albumin, creatinine, calcium and inorganic phosphate (Pi), as well as urinary levels of creatinine and Pi. eGFR was calculated using the creatinine-based Modification of Diet in Renal Disease Study equation [20]. Urinary fractional excretion of phosphate (FEPi) was calculated using the formula: FEPi = (urine phosphate×serum creatinine)/(serum phosphate×urine creatinine)×100. Serum 1,25VitD_3_ levels were measured using a radioimmunoassay, and serum intact parathyroid hormone (PTH) levels were measured using an electrochemiluminescence immunoassay (SRL, Tokyo, Japan). Serum FGF23 and sKL concentrations were measured using enzyme-linked immunosorbent assay (ELISA) kits, as described previously [21]. Levels of renal α-KL expression were evaluated using the renal biopsy samples. The patient characteristics are listed in Table 1. Included in this study were 14 pre-hemodialysis (HD) patients with stage 5 CKD and 43 patients undergoing maintenance HD. Serum samples were collected from the HD patients before HD. All clinical study protocols were approved by the Nara Medical University Ethics Committee (No. 2002-2009). Written informed consent was obtained in all cases, either from the patient or his/her family.

**Table 1:** Clinical characteristics of the study population.

CKD	stage 1	stage 2	stage 3	stage 4	stage 5
Number of patients	61	84	53	20	18
Ages (years)	33±14.6	48.4±15.5	55.1±13.9	56.4±13.7	54.8±20.7
Gender (F/M)	26/35	37/47	20/33	10/10	6/12
Kidney disease					
IgAN	35 (57.3%)	49 (58.3%)	34 (64.2%)	7 (35.0%)	4 (22.2%)
MN	11 (18.1%)	22 (26.2%)	11 (20.8%)	3 (15.0%)	1 (5.6%)
FSGS	2 (3.3%)	2 (2.4%)	3 (5.7%)	4 (20.0%)	0
MCD	13 (21.3%)	10 (11.9%)	5 (9.4%)	0	0
Others	0	1 (1.2%)	0	6 (30.0%)	13 (72.2%)

Abbreviations: CKD, chronic kidney disease; IgAN, IgA nephropathy; MN, membranous glomerulonephritis;

FSGS, focal segmental glomerulosclerosis; MCD, minimal change disease.

Clinical parameters are presented as means ± S.D.

### Immunohistochemical studies

α-KL was immunohistochemically labeled in sections from human renal biopsies and samples of parathyroid gland using KM2076 antibody [22] (1∶50 dilution; a kind gift from Kyowa Hakko Kirin). The labeled protein was visualized using a Dako Envison Kit (Dako, Glostrup, Denmark).

### Recombinant human FGF23

Recombinant human FGF23 harboring two mutations (R176Q and R179Q) found in patients with autosomal dominant hypophosphatemic rickets was used for these studies [23]. A pcDNA3.1 expression vector containing cDNA encoding the FGF23 mutant with a C-terminal His tag was stably expressed in CHO-1 cells and then purified from the conditioned medium using HisTrap™ HP column chromatography (GE Healthcare Japan Corporation, Tokyo, Japan).

### Cell transfection and treatment with FGF23

HEK293 cells plated in 6-well plates were cultured in Dulbecco's modified Eagle's medium containing 10% fetal bovine serum and infected with adeno-α-KL or adeno-Lac Z control vector, as described previously [24]. Two days after transfection, the cells were treated for 30 min with FGF23 (50 ng/ml, 200 ng/ml).

### RNA Extraction, Reverse Transcription and Real-Time RT-PCR

Total cellular RNA was extracted from frozen human renal biopsy specimens or HEK293 cells, after which first-strand cDNA was generated as previously described [25], [26]. For real-time polymerase chain reaction (PCR), 1 µL of each first-strand reaction product was amplified using appropriate primers and the corresponding fluorescent probes for human and murine α-KL (assay IDs: Hs00183100_m1, Mm00502002_m1), human early growth-responsive 1 (Egr-1) (assay ID: Hs 00152928_m1) and human β-actin (assay ID: Hs 00242273_m1). The probes were designed by the Applied Biosystems “Assay-on-Demand” service (Forster City, CA). Human α-KL/β-actin mRNA and Egr-1/β-actin mRNA ratios were calculated for each sample.

### Western blotting

Lysates were prepared from HEK293 cells in lysis buffer, after which western blot analysis was performed as described previously [22] using monoclonal rat anti-α-KL antibody (KM2076)27 (1∶500 dilution).

### Statistical analysis

Statistical analyses were performed using Stat View 5.0 software. Numerical results are expressed as means ± S.D. Student's *t*-test was used for normally distributed variables with two unpaired groups. To compare groups, we used a one-way analysis of variance followed by a post-hoc *t*-test with Fisher's Protected Least Significant Difference adjustment. For variables with a skewed distribution, we used Tukey's Honestly Significant Difference (HSD) post hoc test with Bonferroni's adjustment. Pearson's correlation coefficient was used to assess the relationships between renal α-KL mRNA levels and clinical mineral metabolism parameters and sKL levels. Multiple regression analyses were performed to assess the influence of eGFR, PTH, FGF23, 1,25VitD_3_, corrected calcium, Pi and age on renal α-KL levels; eGFR, renal α-KL, PTH and Pi on serum FGF23 levels; eGFR, FGF23, PTH and Pi on FEPi; eGFR, FGF23, PTH and corrected calcium on serum 1,25VitD_3_ levels; and renal α-KL, eGFR, PTH, FGF23, 1,25VitD_3_, corrected calcium, Pi and age on sKL levels. The null hypothesis was rejected when the P value was less than 0.05.

## Results

### Reductions in renal α-KL correspond to progression of CKD

**Figure 1:**
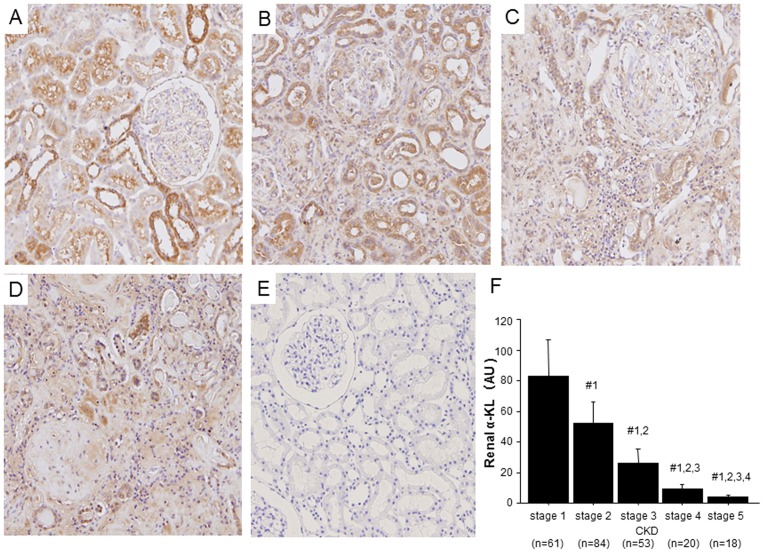
Reduction of renal α-Klotho (α-KL) expression with progression of CKD. (A–D) Representative images showing immunoperoxidase staining of α-KL in renal biopsy sections from CKD patients at (A) stage 1, (B) stage 3, (C) stage 5 and (D) stage 5 HD. (E) Negative control (stage 1 CKD patient treated with rat immunoglobulin instead of rat anti-α-KL antibody). Original magnification, 100×. (F) Renal α-KL mRNA levels. Tukey's Honestly Significant Difference (HSD) post hoc test with Bonferroni's adjustment was used to compare groups: #1, *P*<0.005 vs. stage 1; #2, *P*<0.005 vs. stage 2; #3, *P*<0.005 vs. stage 3; #4, *P*<0.005 vs. stage 4.

α-KL was detected immunohistologically in the kidneys of stage 1 CKD patients, mainly in the distal convoluted tubules (Figure 1A), but α-KL reactivity fell significantly as CKD progressed (Figure 1B–D). Correspondingly, renal α-KL mRNA levels, quantified using real-time PCR, also declined significantly with the progression of renal dysfunction in CKD (Figure 1F).

**Figure 2:**
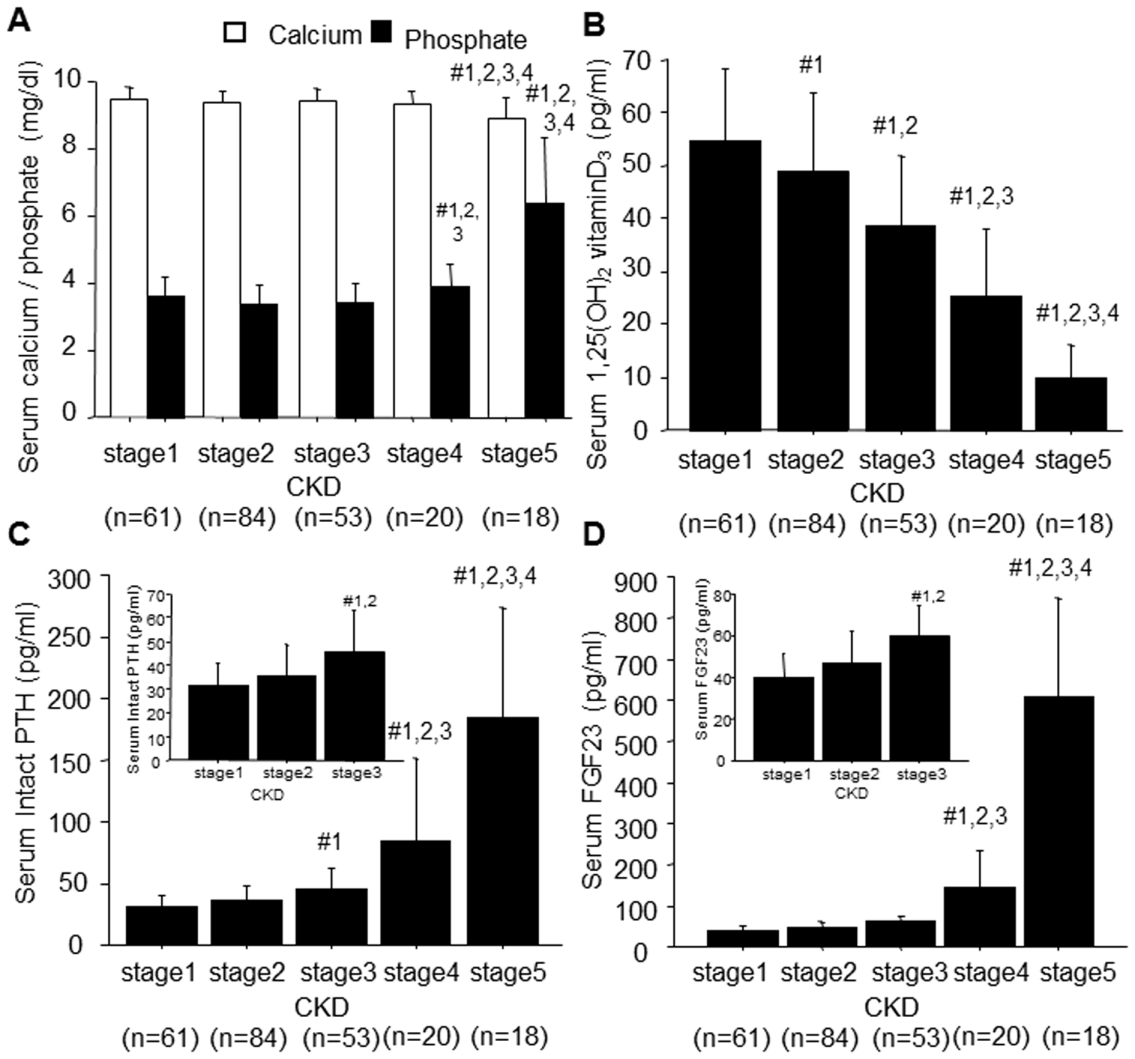
Clinical mineral metabolism parameters in patients with CKD. (A) Serum corrected calcium concentrations (white bars) and inorganic phosphate concentrations (black bars). (B) Serum 1,25VitD_3_ concentrations. (C) Serum intact PTH concentrations. (D) Serum FGF23 concentrations. Data are shown as means ± S.D. Tukey's Honestly Significant Difference (HSD) post hoc test with Bonferroni's adjustment was used to compare groups: #1, *P*<0.005 vs. stage 1; #2, *P*<0.005 vs. stage 2; #3, *P*<0.005 vs. stage 3; #4, *P*<0.005 vs. stage 4.

During the progression of CKD, the decline in renal α-KL levels was followed by reductions in 1,25VitD_3_ and increases in serum FGF23 and intact PTH, which became significant in stage 3 CKD (Figure 2B–D). On the other hand, serum Pi levels remained within the normal range until stage 4, and serum calcium levels remained within the normal range until stage 5 (Figure 2A).

Examination of several clinical mineral metabolism parameters showed significant correlations between the renal levels α-KL mRNA and serum levels of calcium (r = 0.198, P = 0.0022), Pi (r = −0.283, P<0.0001), 1,25VitD_3_ (r = 0.549, P<0.0001), FGF23 (r = −0.461, P<0.0001) and intact PTH (r = −0.469, P<0.0001) across all patients (Figure S1A–E). There was also a significant correlation between renal α-KL expression and eGFR (r = 0.889, P<0.001) (Figure S1F). Multiple regression analysis revealed renal α-KL levels to significantly and positively correlate with only eGFR (β = 0.928, P<0.001) as an independent contributing factor across all patients (R^2^ = 0.792, P<0.0001) (Table 2).

**Table 2:** Multiple regression analysis^A^ of renal α-KL in CKD patients.

Independent variables	β^B^	*P* value
1,25(OH)_2_ vitamin D_3_	−0.014	0.7503
Intact PTH	0.024	0.6313
FGF23	0.045	0.4908
Serum corrscted calcium	0.002	0.9496
Serum inorganic phosphate	−0.002	0.9715
Age	−0.015	0.6822
eGFR^a^	0.928	<0.0001

A Adjusted coefficient of determination (R^2^); R^2^ = 0.792 , *P*<0.0001.

B Standard partial regression coefficient.

Abbreviations: α-KL, α-klotho; PTH, parathyroid hormone; FGF23, fibroblast growth factor 23;

eGFR, estimated glomerular filtration rate.

a eGFR was calculated using the creatinine-based Modification of Diet in Renal Disease Study Equation.

### Association between serum FGF23 and urinary fractional excretion ratio of phosphate (FEPi) and serum 1,25VitD_3_ in CKD patients

**Figure 3:**
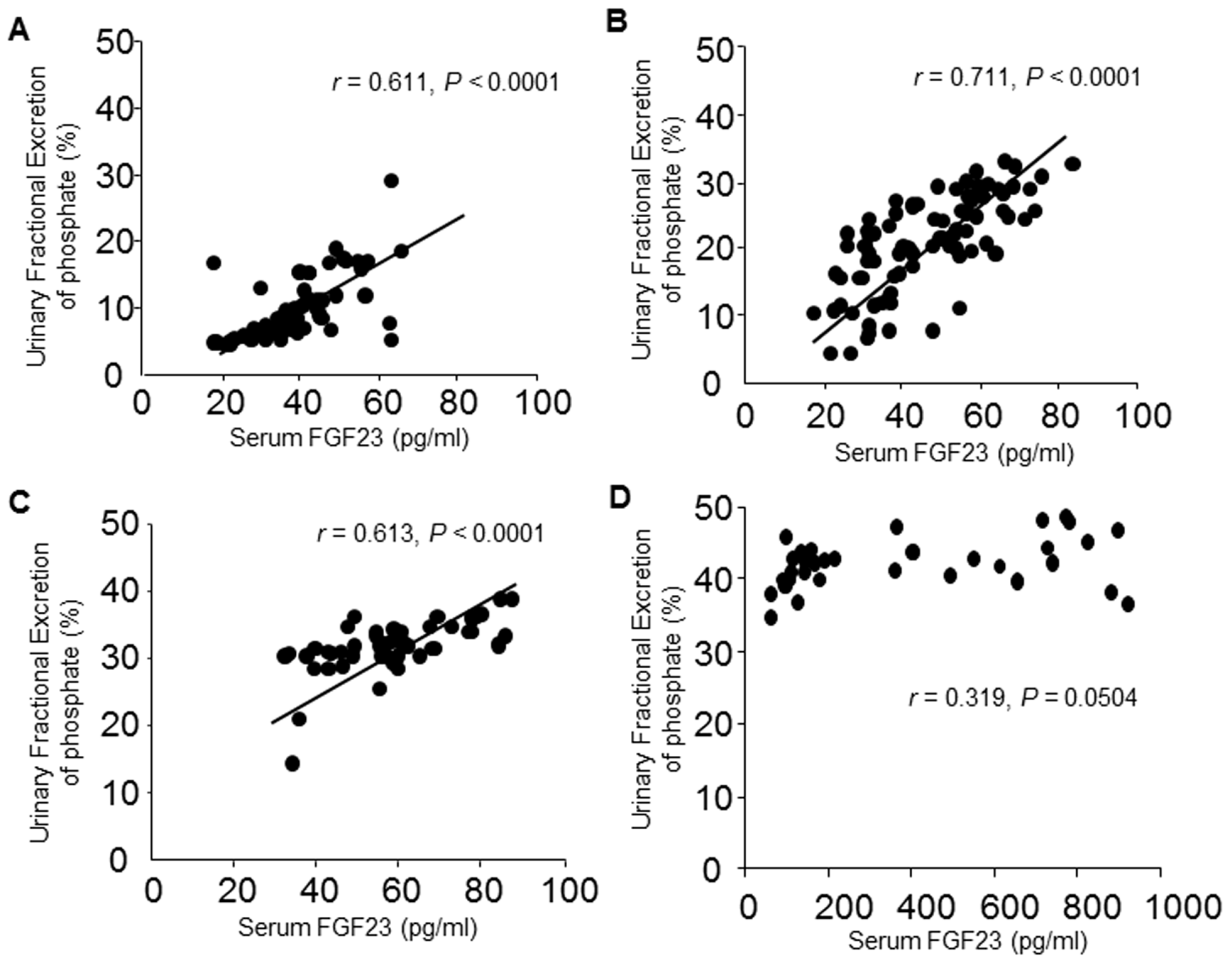
Correlations between serum FGF23 and urinary fractional excretion of phosphate (FEPi) in CKD patients. (A–D) Serum FGF23 concentration plotted against FEPi in CKD patients at stage 1 (A), 2 (B), 3 (C) and 4–5 (D). Correlations were evaluated using Pearson's correlation coefficient.

We next studied whether, as CKD progresses, the kidney becomes increasingly resistant to FGF23-induced urinary phosphate excretion and 1,25VitD_3_ production is suppressed, and whether there is a related loss of renal α-KL. We analyzed the associations between serum FGF23 and FEPi and serum 1,25VitD_3_ in CKD patients at all stages of the disease. Univariate analysis showed that there is a significant positive correlation between serum FGF23 levels and FEPi in CKD patients at stages 1 (r = 0.611, P<0.0001), 2 (r = 0.711, P<0.0001) and 3 (r = 0.613, P<0.0001), but not at stages 4–5 (r = 0.319, P = 0.0504) (Figure 3A–D). Then to further explore the influence of FGF23 on FEPi in early CKD, we performed a multiple regression analysis of FEPi using eGFR and serum FGF23, intact PTH and Pi levels as explanatory factors across CKD patients at stages 1, 2 and 3. FEPi was significantly and positively correlated with FGF23 (β = 0.401, P<0.0001) and inversely correlated with eGFR (β = −0.679, P<0.0001) as independent contributing factors (R^2^ = 0.830, P<0.0001) (Table S1).

**Figure 4:**
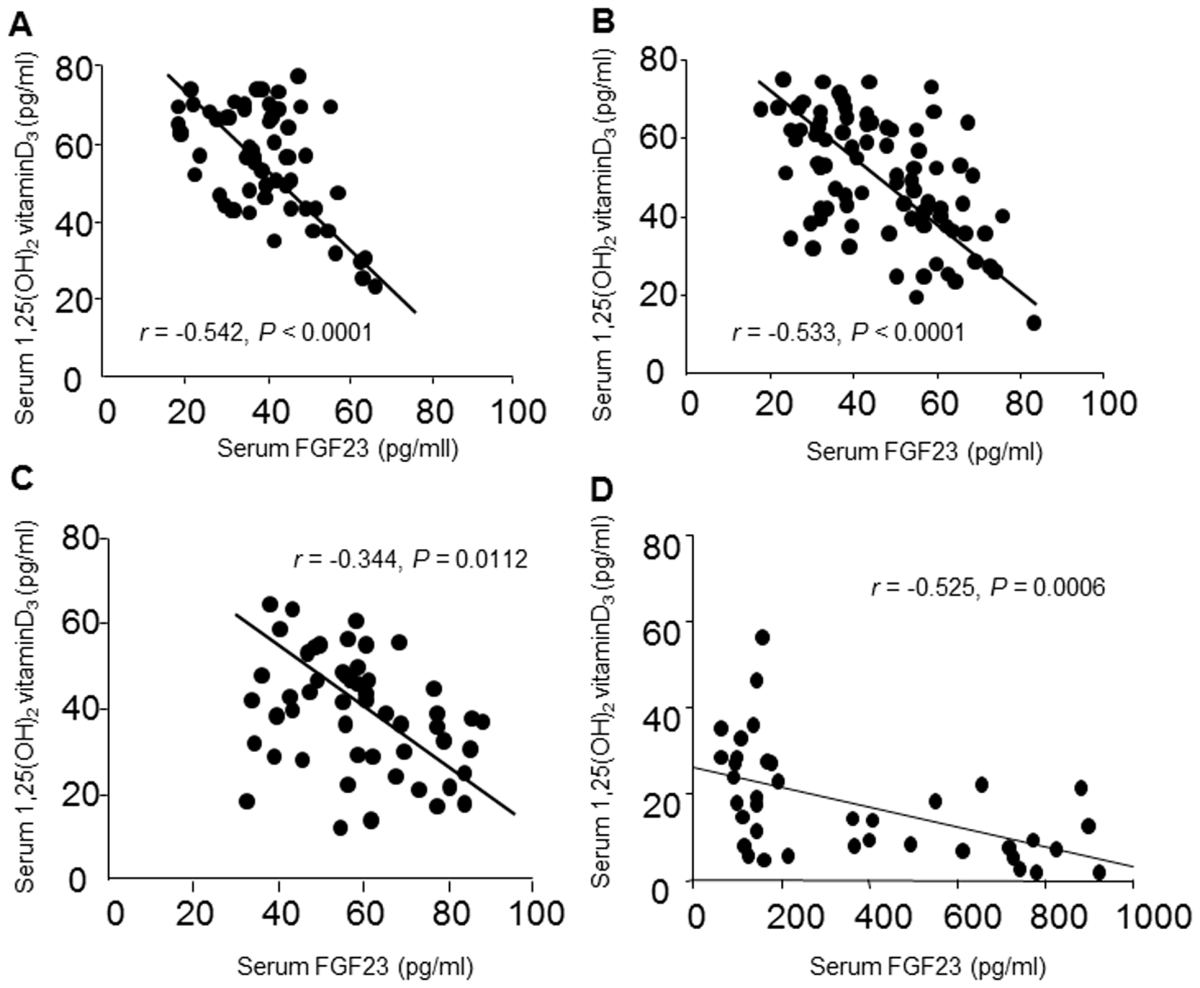
Correlation between serum FGF23 and 1,25VitD3 in CKD patients. (A–D) Serum FGF23 concentration plotted against 1,25VitD_3_ concentration in CKD patients at stage 1 (A), 2 (B), 3 (C) and 4–5 (D). Correlations were evaluated using Pearson's correlation coefficient.

On the other hand, univariate analyses showed that serum FGF23 levels significantly and inversely correlate with serum 1,25VitD_3_ levels in CKD patients at stages 1 (r = −0.542, P<0.0001), 2 (r = −0.533, P<0.0001), 3(r = −0.344, P = 0.0112) and 4–5 (r = −0.525, P = 0.0006) (Fig. 4A–D). In addition, when we focused on patients with stage 1, 2 or 3 CKD, we found that the association between serum FGF23 and 1,25VitD_3_ gradually weakened with disease progression. Multiple regression analysis of 1,25VitD_3_ using eGFR and serum FGF23, intact PTH and calcium as explanatory factors across CKD patients at stages 1, 2 and 3 revealed 1,25VitD_3_ levels to be significantly and inversely correlated with FGF23 (β = −0.493, P<0.0001) and positively correlated with eGFR (β = 0.213, P = 0.0028) as independent contributing factors (R^2^ = 0.310, P<0.0001) (Table S2). These results indicate that FGF23 can induce both elevations in FEPi and reductions in serum 1,25VitD_3_ in early CKD patients, despite a gradual decline in renal α-KL levels.

### Association between serum FGF23 with renal α-KL in CKD patients

**Figure 5:**
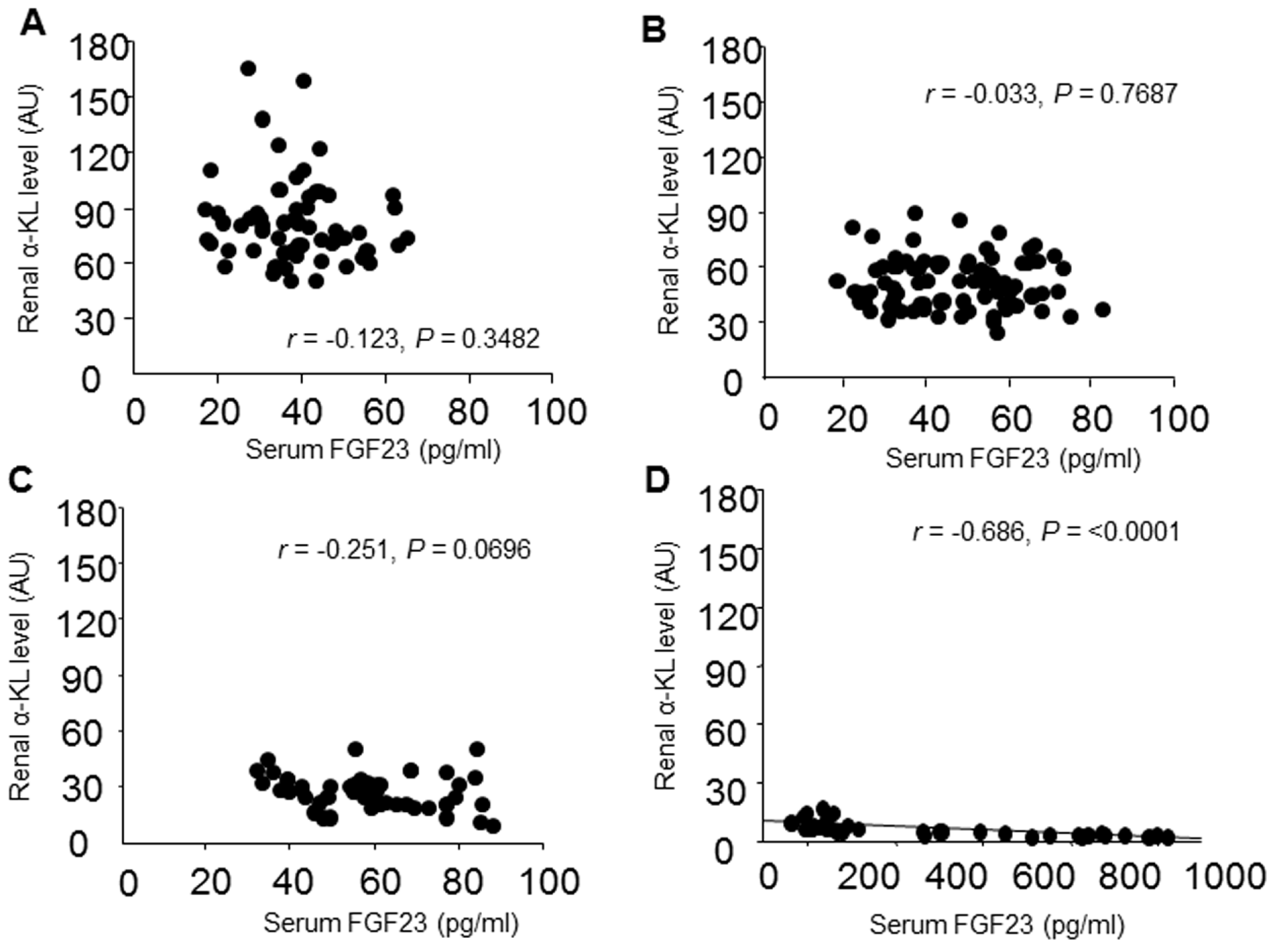
Correlation between serum FGF23 and renal α-Klotho (α-KL) in CKD patients. (A–D) Serum FGF23 concentration plotted against renal α-KL level in CKD patients at stage 1 (A), 2 (B), 3 (C) and 4–5 (D). Correlations were evaluated using Pearson's correlation coefficient.

To determine whether the reduction in renal α-KL is a primary factor contributing to the increase in FGF23 secretion seen in early CKD patients, we assessed the association between serum FGF23 and renal α-KL levels. Univariate analysis showed that there is not a significant correlation between serum FGF23 and renal α-KL levels in CKD patients at stage 1 (r = −0.123, P = 0.3482), 2 (r = −0.033, P = 0.7687) or 3 (r = −0.251, P = 0.0696), but there is a significant inverse correlation at stages 4–5 (r = −0.686, P<0.0001) (Figure 5A–D). Then to examine the affects of increasing serum FGF23 levels in early CKD, multiple regression analysis of FGF23 was performed using eGFR, renal α-KL, serum intact PTH and Pi as explanatory factors across early CKD patients (stages 1–3). In these patients, serum FGF23 levels correlated significantly and inversely with eGFR (β = −0.382, P = 0.0013) and correlated positively with Pi levels (β = 0.168, P = 0.0085) as independent contributing factors (R^2^ = 0.256, P<0.0001) (Table 3). Similarly, in advanced CKD patients (stages 4–5), multiple regression analysis showed serum FGF23 levels to correlate significantly and inversely with eGFR (β = −0.574, P = 0.0156) and to correlate positively with Pi levels (β = 0.381, P = 0.0051) as independent contributing factors (R^2^ = 0.813, P<0.0001), but not with renal α-KL levels (Table 4). These results suggest that loss of renal α-KL is not a primary factor enhancing FGF23 secretion in CKD patients.

**Table 3:** Multiple regression analysis^A^ of serum FGF23 levels in early CKD patients (stages 1–3).

Independent variables	β^B^	*P* value
Intact PTH	0.103	0.1382
eGFR^a^	−0.382	0.0013
Serum Pi	0.168	0.0085
Renal α-KL	−0.083	0.4582

A Adjusted coefficient of determination (R^2^); R^2^ = 0.256 , *P*<0.0001.

B Standard partial regression coefficient.

Abbreviations: α-KL, α-klotho; PTH, parathyroid hormone; eGFR, estimated glomerular filtration rate,

FGF23, fibroblast growth factor 23; Pi, inorganic phosphate.

a eGFR was calculated using the creatinine-based Modification of Diet in Renal Disease Study Equation.

**Table 4:** Multiple regression analysis^A^ of serum FGF23 levels in advanced CKD patients (stages 4–5).

Independent variables	β^B^	*P* value
Intact PTH	0.061	0.5424
eGFR^a^	−0.574	0.0156
Serum Pi	0.381	0.0051
Renal α-KL	0.044	0.8012

A Adjusted coefficient of determination (R^2^); R^2^ = 0.813, *P*<0.0001.

B Standard partial regression coefficient.

Abbreviations: α-KL, α-klotho; PTH, parathyroid hormone; eGFR, estimated glomerular filtration rate,

FGF23, fibroblast growth factor 23; Pi, inorganic phosphate.

a eGFR was calculated using the creatinine-based Modification of Diet in Renal Disease Study Equation.

### The effect of *α-KL* expression on FGF23-induced up-regulation of *Egr-1* expression in HEK293 cells

**Figure 6:**
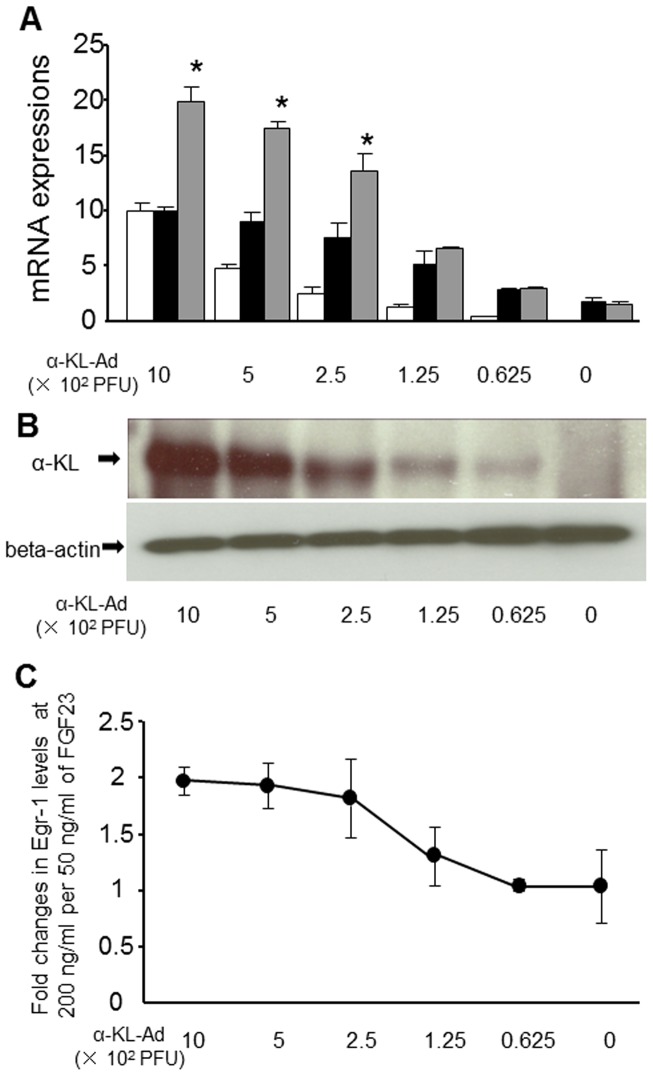
Effect of *α-KL* expression on FGF23-induced up-regulation of *Egr-1* expression in HEK293 cells. HEK293 cells were transfected with the indicated dose of adenovirus carrying the *α-KL* (α-KL-Ad) or *Lac Z* gene (control). (A) Expression of Egr-1 (black and gray bars) and α-KL (white bars) mRNA. The transfectant cells were incubated for 30 min with FGF23 at 50 ng/ml (black bars) or 200 ng/ml (gray bars). (B) Western blotting of α-KL. (C) Fold changes in Egr-1 mRNA levels induced by FGF23 at 200 ng/ml, as compared to 50 ng/ml. Data are shown as means ± S.D. Student's t-test was used to compare groups. *P<0.05 vs. HEK293 cells co-cultured with 50 ng/ml FGF23.

To further clarify whether a gradual decline in renal α-KL leads to resistance to FGF23 signaling, we transfected HEK293 cells with different amounts of adenoviral vector encoding α-KL, and then analyzed FGF23-induced *Egr-1* expression in the transfectants. FGF23 reportedly up-regulates *Egr-1* gene expression in cultured cells expressing *α-KL* at different levels [3]. We found that FGF23-induced *Egr-1* expression gradually declined in proportion to *α-KL* expression (Figure 6A, B). In addition, when we increased FGF23 by about 4× in HEK293 cells transfected with *α-KL*, *Egr-1* expression increased about 2×, but the increase in *Egr-1* expression was dramatically and dose-dependently attenuated when *α-KL* expression was reduced by approximately 80% (Figure 6C). We also found that *Egr-1* expression did not significantly increase in HEK293 cells in the absence of α-KL, even when FGF23 was increased (Figure 6A, B). In this experiment, HEK293 cells transfected with Lac Z gene served as the control.

### Association between serum sKL and renal α-KL in CKD patients

**Figure 7:**
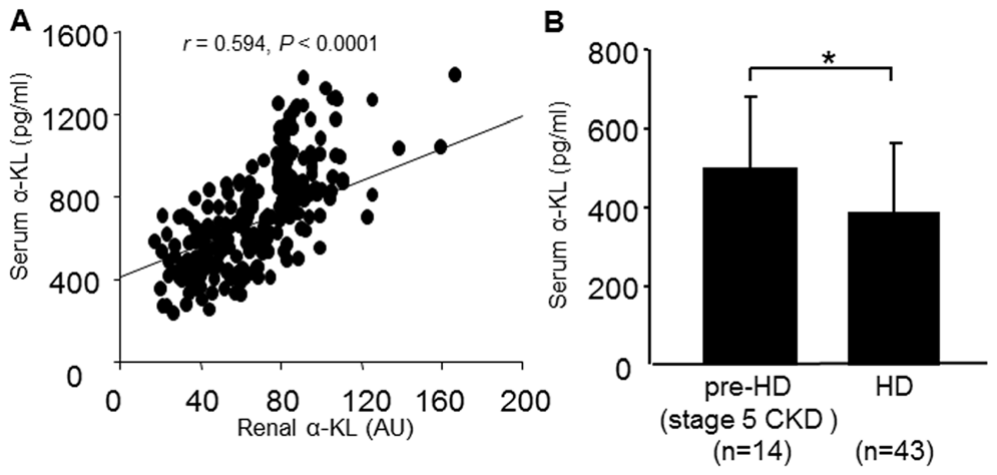
Serum soluble α-Klotho (α-KL) levels in CKD patients. (A) Correlation between the serum soluble α-KL concentration and renal α-KL mRNA level in CKD patients. (B) Serum soluble α-KL concentration in pre-HD stage 5 CKD patients and hemodialysis (HD) patients. Correlations were evaluated using the Pearson's correlation coefficient. Data are shown as means ± S.D. Student's *t*-test was used to compare two unpaired groups. *P<0.01.

To determine whether the gradually developing renal α-KL deficiency affects serum sKL levels in CKD patients, we assessed the association between serum sKL and renal α-KL levels. We found that serum sKL declined significantly with falling renal α-KL in CKD patients (r = 0.594, P<0.0001) (Figure 7A). Moreover, multiple regression analysis of sKL using age, eGFR, renal α-KL, FGF23, intact PTH, 1,25VitD_3,_ calcium and Pi levels as explanatory factors showed that serum sKL levels significantly correlate with renal α-KL (β = 0.803, P<0.001) as an independent contributing factor (R^2^ = 0.382, P<0.0001) across all CKD patients (Table 5). In addition, when we analyzed sKL levels in patients on maintenance HD, who expressed very little renal α-KL (Figure 1D), we found their serum sKL levels to be significantly lower than in pre-HD patients with stage 5 CKD (HD, 383.1±179.9 pg/ml; pre-HD, 495.6±181.9 pg/ml, P<0.05) (Figure 7B). These results suggest sKL levels could be a useful marker of renal α-KL levels.

**Table 5:** Multiple regression analysis^A^ of serum soluble α-KL in CKD patients.

Independent variables	β^B^	*P* value
Renal α-KL	0.803	<0.0001
1,25(OH)_2_ vitamin D_3_	−0.085	0.2525
Intact PTH	0.01	0.9045
FGF23	0.05	0.6543
Serum corrscted calcium	−0.008	0.8933
Serum inorganic phosphate	−0.047	0.6033
Age	0.105	0.0943
eGFR^a^	−0.218	0.1125

A Adjusted coefficient of determination (R^2^); R^2^ = 0.382 , *P*<0.0001.

B Standard partial regression coefficient.

Abbreviations: α-KL, α-klotho; PTH, parathyroid hormone; FGF23, fibroblast growth factor 23;

eGFR, estimated glomerular filtration rate.

a eGFR was calculated using the creatinine-based Modification of Diet in Renal Disease Study Equation.

## Discussion

Our findings in the present study show that renal α-KL expression declines with declining eGFR and that it correlates significantly with mineral metabolite levels. This suggests mineral metabolism disorders are associated with renal α-KL deficiency in CKD. In addition, multiple regression analysis clearly showed that renal dysfunction is the most important factor contributing to the reduction in renal α-KL expression in CKD patients. On the other hand, we also found that FGF23 begins to increase early during the progression of CKD, and that FGF23 levels were high when renal α-KL expression was low. We therefore speculate that within the kidneys of CKD patients, the α-KL deficiency leads to FGF23 resistance, which drives compensatory increases in FGF23 secretion. Consistent with that idea, α-KL^+/−^ mice showed significantly higher plasma FGF23 levels than α-KL^+/+^ mice [3]. Unexpectedly, when we focused on early CKD patients (stages 1, 2 and 3), univariate analysis indicated that serum FGF23 levels did not correlate significantly with renal α-KL levels, and multiple regression analysis clearly showed that eGFR and serum Pi were significant independent determinants of serum FGF23 levels, but renal α-KL was not. In short, the increase in FGF23 seen in early CKD is probably induced by phosphate hoarding due to impaired urinary phosphorus excretion related to nephron loss, not to FGF23 resistance related to α-KL deficiency.

We then investigated the effect of renal α-KL deficiency on FGF23-induced phosphaturia and suppression of renal 1,25VitD_3_ production. We confirmed that FGF23 was a significant determinate reducing serum 1,25VitD_3_ levels and enhancing FEPi in early CKD patients, even though renal α-KL was falling. This is consistent with a scenario in which an increase in FGF23 can mitigate phosphate retention and suppress 1,25VitD_3_ production, thereby normalizing serum Pi levels in early CKD. In addition, the suppression of 1,25VitD_3_ would lead to a secondary increase in PTH. Thus early CKD patients do not exhibit FGF23 resistance, despite a fall in renal α-KL levels.

When we focused on advanced CKD patients (stages 4–5), multiple regression analysis showed that FGF23 was not a significant independent determinant of FEPi or serum 1,25VitD_3_. Indeed, FEPi was not significantly affected by increases in serum FGF23, which led to higher serum Pi levels. We therefore speculate that resistance of the remaining functional nephrons to FGF23, induced by the decline in α-KL expression, may contribute to a reduction in urinary phosphate excretion. Notably, when we focused on patients with serum FGF23 levels greater than 500 pg/ml (n = 12), we found that FEPi values plateaued or declined slightly, despite marked increases in serum FGF23 (Figure 3D). This suggests the observed increases in FGF23 cannot sufficiently suppress the reabsorption of urinary phosphate by the remaining functional nephrons. Moreover, we found that there was a significant association between FEPi and renal α-KL (r = 0.614, P = 0.0319), but not eGFR (r = 0.238, P = 0.4664). Indeed, we found that in advanced CKD patients, renal α-KL levels fell significantly to about 20% of the levels seen in stage 1 CKD patients. This suggests that with an approximately 80% reduction in α-KL, renal FGF23 resistance may develop. Consistent with that idea, when α-KL expression was reduced by approximately 80% in HEK293 cells, we detected a marked threshold effect on FGF23, leading to significant attenuation in FGF23-induced *Egr-1* expression. In other words, when CKD advances to a point where renal α-KL levels are insufficient to support FGF23 signaling, FGF23-mediated increases in FEPi are impaired with resultant increases in serum Pi. Moreover, multiple regression analysis showed serum Pi to be a significant independent determinant of FGF23 levels in patients with advanced CKD.

sKL is thought to be produced through alternative splicing of the α-KL transcript or through release of the extracellular domain of membrane-anchored α-KL [15], [16], [17], [18]. RT-PCR analyses have shown that α-KL is expressed in a variety of tissues, but the highest expression is in the kidney [1], [27]. We therefore predicted that serum sKL levels would gradually decline in proportion to renal α-KL expression as CKD progressed. As expected, multiple regression analysis clearly showed renal α-KL to be the most important factor contributing to the reduction in serum sKL levels in CKD patients. In addition, serum sKL was significantly diminished in HD patients, whose kidneys express much less α-KL than stage 5 CKD patients. These results indicate that the serum sKL level could be a useful biomarker of renal α-KL expression.

In summary, we found that renal dysfunction initially induces a reduction in renal α-KL expression, which in turn reduces circulating sKL levels. This suggests the serum sKL concentration may be a useful marker of the renal α-KL level. We also found that secretion of FGF23 into the circulation is enhanced by renal failure-related Pi hoarding at early stages of CKD. The resultant rise in FGF23 increased FEPi and reduced 1,25VitD_3_ levels via FGF23-α-KL signaling. This would in turn lead to normalization of serum Pi levels, despite falling renal α-KL expression. In advanced CKD, by contrast, levels of α-KL are not sufficient to support renal FGF23-α-KL signaling, so FGF23 cannot compensate for the renal failure-induced Pi retention. Consequently, serum Pi is elevated, which would stimulate further increases in FGF23 secretion. It is thus important to assess renal α-KL expression in CKD patients for appropriate management of serum FGF23 levels.

## Supporting Information

Table S1 Multiple regression analysisA of urinary fractional excretion of phosphate (FEPi) in CKD patients at stages 1, 2 and 3.
(DOCX)

Table S2 Multiple regression analysisA of serum 1,25(OH) 2 vitamin D3 levels in CKD patients at stages 1, 2 and 3.
(DOCX)

Figure S1 **Correlation between renal α-KL mRNA levels and clinical mineral metabolism parameters and eGFR in CKD patients.** (A) Renal α-KL mRNA levels are plotted against serum concentrations of corrected calcium, (B) inorganic phosphate, (C) 1,25(OH)2 vitaminD3, (D) FGF23 and (E) intact PTH, as well as (F) eGFR in CKD patients. Correlations were evaluated using Pearson's correlation coefficient.
(TIF)
