# Supplementary material for: Reduced Renal α-Klotho Expression in CKD Patients and Its Effect on Renal Phosphate Handling and Vitamin D Metabolism
Source: PLoS One. 2014 Jan 23;9(1):e86301. doi: 10.1371/journal.pone.0086301 (PMC3900516; doi:10.1371/journal.pone.0086301)
Supplement: Table S1 — Multiple regression analysisA of urinary fractional excretion of phosphate (FEPi) in CKD patients at stages 1, 2 and 3. (DOCX) [file pone.0086301.s001.docx]

**Table S1. Multiple regression analysis^A^ of urinary fractional excretion of phosphate (FEPi) in CKD patients at stages 1, 2 and 3**

| independent variables | β^B^ | *P* value |
| --- | --- | --- |
| eGFR^a^ | -0.679 | <0.0001 |
| FGF23 | 0.401 | <0.0001 |
| Intact PTH | -0.055 | 0.1014 |
| Serum Pi | 0.052 | 0.0921 |

^A^Adjusted coefficient of determination (R^2^ ); R^2^ =0.830, *P* <0.0001.

^B^Standard partial regression coefficient

Abbreviations: FGF23, fibroblast growth factor 23; PTH, parathyroid hormone;

eGFR, estimated glomerular filtration rate; Pi, inorganic phosphate.

^a^ eGFR was calculated using the creatinine-based Modification of Diet in Renal Disease Study Equation.
