# Supplementary material for: Reduced Renal α-Klotho Expression in CKD Patients and Its Effect on Renal Phosphate Handling and Vitamin D Metabolism
Source: PLoS One. 2014 Jan 23;9(1):e86301. doi: 10.1371/journal.pone.0086301 (PMC3900516; doi:10.1371/journal.pone.0086301)
Supplement: Table S2 — Multiple regression analysisA of serum 1,25(OH) 2 vitamin D3 levels in CKD patients at stages 1, 2 and 3. (DOCX) [file pone.0086301.s002.docx]

**Table S2. Multiple regression analysis^A^ of serum 1,25(OH) _2_ vitamin D_3_ levels in CKD patients at stages 1, 2 and 3**

| Independent variables | β^B^ | *P* value |
| --- | --- | --- |
| FGF23 | -0.493 | <0.0001 |
| Intact PTH | 0.082 | 0.2038 |
| eGFR^a^ | 0.213 | 0.0028 |
| Serum corrected calcium | -0.054 | 0.3662 |

^A^Adjusted coefficient of determination (R^2^ ); R^2^ =0.310 , *P* <0.0001.

^B^Standard partial regression coefficient

Abbreviations: FGF23, fibroblast growth factor 23; PTH, parathyroid hormone;

eGFR, estimated glomerular filtration rate.

^a^ eGFR was calculated using the creatinine-based Modification of Diet in Renal Disease Study Equation.
