# Supplementary figures and images for: Reduced Renal α-Klotho Expression in CKD Patients and Its Effect on Renal Phosphate Handling and Vitamin D Metabolism
Source: PLoS One. 2014 Jan 23;9(1):e86301. doi: 10.1371/journal.pone.0086301 (PMC3900516; doi:10.1371/journal.pone.0086301)

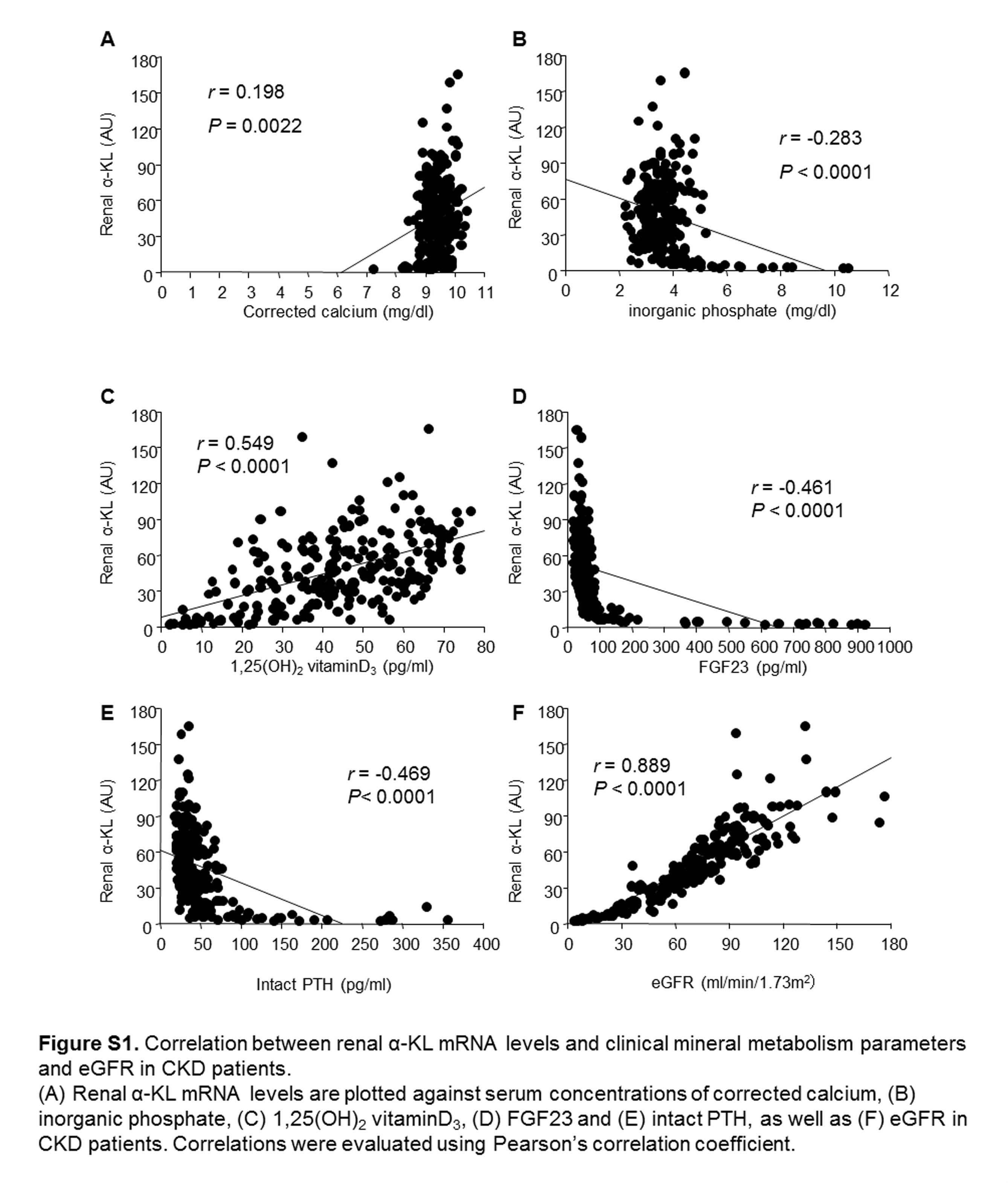

Supplement: Figure S1 — Correlation between renal α-KL mRNA levels and clinical mineral metabolism parameters and eGFR in CKD patients. (A) Renal α-KL mRNA levels are plotted against serum concentrations of corrected calcium, (B) inorganic phosphate, (C) 1,25(OH)2 vitaminD3, (D) FGF23 and (E) intact PTH, as well as (F) eGFR in CKD patients. Correlations were evaluated using Pearson's correlation coefficient. (TIF) [file pone.0086301.s003.tif]
